# Utility of Obtaining Follow-Up Blood Cultures in Gram-Negative Bacterial Bloodstream Infection Among Patients With Hematologic Malignancies

**DOI:** 10.7759/cureus.73915

**Published:** 2024-11-18

**Authors:** Nitya Dhanaraj, Kelly Rowe, Carlo Foppiano Palacios

**Affiliations:** 1 Medicine, Cooper Medical School of Rowan University, Camden, USA

**Keywords:** blood culture shortage, gram-negative bacteremia, infections in neutropenic fever, persistent bacteremia, repeat blood culture

## Abstract

Background: There is an ongoing debate on the benefit of obtaining repeat blood cultures for Gram-negative bacterial bloodstream infections (GNBSI). However, there is a scarcity of data concerning patients with hematologic malignancies. We sought to assess the utility of obtaining follow-up blood cultures (FUBC) in GNBSI among patients with hematologic malignancies.

Methods: A retrospective chart review was conducted to identify all admitted patients with hematologic malignancies with GNBSI from 2018 to 2021 at a tertiary academic medical center. We collected demographics, cancer history and treatment, microbiology and antibiotic use, and clinical course. Descriptive statistics were used.

Results: A total of 46 episodes of GNBSI among 38 patients were included. The median age was 61.5 years, 63% were male, 50% were White, and 16% were Latinx. Most patients had acute myeloid leukemia (60%), and the most common chemotherapy regimen was cytarabine or nelarabine regimens (35%). *Klebsiella pneumoniae* was responsible for 37% of the GNBSI cases, in the setting of long-term central venous catheter use (65%) and gastrointestinal sources (50%). FUBCs were collected among almost all patients (98%). Only three cases (7%) had positive FUBC: one had a delay in appropriate therapy, another had a lack of source control, and the last case did not have a source identified. Most patients were treated with beta-lactams (52%) with duration 8-14 days (52%). 22% were admitted to ICU and 9% died during their hospitalization.

Conclusion: We found a few cases of positive FUBC. Routine FUBC may not be appropriate for all GNBSI patients with hematological malignancies, particularly during the current blood culture bottle shortage.

## Introduction

Gram-negative bacterial bloodstream infections (GNBSIs) are associated with a high mortality rate of 13-20% [1,2]. The optimal management of patients with GNBSI is unclear, particularly among immunocompromised patients. Acquiring follow-up blood cultures (FUBCs) is standard practice to document clearance of infection among patients with *Staphylococcus aureus* bloodstream infection [3]. FUBCs ensure source control has been achieved and aid in determining the appropriate duration of antimicrobial therapy [3,4]. The utility of FUBC in GNBSI, however, remains unclear. Several observational studies and a recent meta-analysis suggest that obtaining FUBC for GNBSI may be associated with a mortality benefit, although the mechanism for this potential benefit is uncertain [5]. Alternatively, FUBC may yield false positive results due to blood culture contaminants, leading to hospital stays, unnecessarily prolonged use of antimicrobials, and additional diagnostic tests [6]. Limited data are available among patients with hematologic malignancies with GNBSI, particularly with the length and severity of neutropenia and complexities from their chemotherapy regimen. We sought to evaluate the usefulness of obtaining FUBC in GNBSI among patients with hematological malignancies.

## Materials and methods

Study population

We conducted a retrospective chart review at Cooper University Hospital, a large urban academic medical center affiliated with MD Anderson Cancer Center at Cooper in Camden, New Jersey. Eligible participants were identified through the electronic medical records if they had a positive blood culture for Gram-negative bacteria and a diagnosis of hematologic malignancy. We included all Gram-negative bacterial bloodstream infection cases among hospitalized patients with hematologic malignancies from 2018 to 2021. We excluded patients younger than 18 years old if they died within 72 hours of initial blood culture collection, and cases with prior positive blood cultures with the same organism within the prior two weeks. A 72-hour exclusion window was implemented to avoid the inclusion of patients who may have died before clinical teams had an opportunity to acquire follow-up blood cultures. This study was approved by the Institutional Review Board at Cooper University Healthcare.

Data collection

We collected demographics, risk factors for infection, clinical characteristics (Charlson Comorbidity Index, medical comorbidities), oncologic diagnosis and treatment, presence of a central venous catheter, microbiological culture and antibiotic sensitivity, clinical history, and antibiotic treatment from electronic health records. Severe neutropenia was defined as an absolute neutrophil count (ANC) of less than 500 cells/mm³. Antimicrobial resistance patterns were specifically examined for AmpC β-lactamase, extended-spectrum β-lactamase (ESBL), and carbapenem resistance. Furthermore, we obtained data on complications of GNBSI: ICU admission, use of systemic vasopressors, mechanical intubation, hospital length of stay, readmissions, and mortality.

Outcomes and statistical analysis

Our primary outcome was the number of cases with positive FUBC. Secondary outcomes included the association between acquisition of FUBC and mortality, and death at 30 and 90 days from discharge. We summarized demographic and clinical data using descriptive statistics, with medians and interquartile ranges (IQR) determined for continuous variables and counts and percentages for categorical variables. Secondary outcomes were planned to be analyzed using Fisher’s exact test. A p-value below 0.05 was deemed statistically significant. Statistical testing was performed using R version 4.0.2.

## Results

Patient characteristics

A total of 46 episodes of GNBSI among 38 patients were included. Median age was 61.5 (IQR 19.5) years, 63.2% (N=24/38) were male, 50.0% (N=19/38) were White, and 15.8% (N=6/38) were Latinx (Table 1). Most patients had AML (N=15/38, 39.5%) and the most common medical comorbidity was diabetes (N=7, 18.4%). The median Charlson Comorbidity Index was 5 (IQR 2).

**Table 1:** Patient demographics (N=38) *Other B-cell lymphomas included diffuse large cell lymphoma (DLBCL), Burkitt lymphoma, and mantel cell lymphoma.

	N	%
Age – median (IQR)	61.5 (19.5)	
Sex		
Female	14	36.8
Male	24	63.2
Race	1	2.6
Asian	9	23.7
Black	19	50
White	9	23.7
Other		
Latinx ethnicity	6	15.8
Hematologic malignancy		
Acute myeloid leukemia	15	39.5
Acute lymphoblastic leukemia	9	23.7
Chronic leukemia	2	5.3
CNS lymphoma	3	7.9
Other B-cell lymphoma^*^	9	23.7
Medical comorbidities		
Diabetes	7	18.4
HIV	1	0
End-stage renal disease	0	2.6
Cirrhosis	2	5.3

Clinical features of infection

Most cases occurred while patients were on active chemotherapy with cytarabine or nelarabine regimens (N=16/46, 34.8%) (Table 2). Most patients had severe neutropenia with ANC <500 (N=39/46, 84.8%) at the time of initial positive blood cultures, and few episodes were hospital-acquired (N=18/46, 39.1%). *Klebsiella pneumoniae* was the most frequently isolated Gram-negative bacteria (N=17/46, 37.0%). Most cases occurred in the setting of long-term central venous catheters (N=40/46, 65.2%) and originated from gastrointestinal sources (N=23/46, 50.0%). Resistance patterns were not commonly identified, including AmpC β-lactamase (N= 5/46, 10.9%), extended-spectrum β-lactamase (N=3/46, 8.7%), and carbapenem-resistant *K. pneumoniae* (KPC) in one case (2.2%).

**Table 2:** Features of GNBSI events (N=46) ANC: absolute neutrophil count; ESBL: extended-spectrum β-lactamase; TMP/SMX: Trimethoprim/Sulfamethoxazole *CHOP regimens include cyclophosphamide, doxorubicin, vincristine, and prednisone. ^#^CVAD regimens include cyclophosphamide, doxorubicin, vincristine, cytarabine, methotrexate, and dexamethasone. ^$^Other regimens included methotrexate only, all-trans retinoic acid, rituximab and bendamustine, enasidenib, pomalidomide, and trial medications. ^£^Other bacteria included Stenotrophomonas maltophilia and Burkholderia cepacia. ^¥^Other antibiotics included aminoglycosides and tetracyclines.

	N	%
Active chemotherapy regimen		
Azacitidine	3	6.5
Blinatumomab	2	4.3
CHOP-regimens^*^	9	19.6
CVAD-regimens^#^	7	15.2
Cytarabine or nelarabine regimens	16	34.8
Other regimen^$^	5	10.8
None	3	6.5
ANC at the time of positive blood cultures		
ANC <500 cells/µL	39	84.8
ANC ≥500 cells/µL	7	15.2
Location acquired		
Community	28	60.9
Hospital	18	39.1
Bacteria isolated		
*Enterobacter cloacae*	3	6.5
*Escherichia coli*	15	32.6
*Klebsiella pneumoniae*	17	37
*Pseudomonas aeruginosa*	9	19.6
*Serratia marcescens*	2	4.3
Other bacteria^£^	2	4.3
Central venous catheter	30	65.2
Source of bloodstream infection		
Central line	5	10.9
Gastrointestinal	23	50
Skin	1	2.2
Urinary	7	15.2
Unknown	10	21.7
Antibiotic resistance patterns		
AmpC β-lactamases	5	10.9
Carbapenemases	1	2.2
Ciprofloxacin resistant	5	10.9
ESBL	4	8.7
Appropriate initial antibiotics	40	87.8
Blood cultures		
Follow-up blood culture obtained	45	97.8
Positive repeat blood culture	3	6.7
Final antibiotic		
Beta lactam	24	52.2
Fluoroquinolone	17	37
Metronidazole	8	17.4
TMP/SMX	4	8.7
Other antibiotics^¥^	2	4.4
Length of antibiotics		
7 days or less	3	6.5
8 to 14 days	24	52.2
15 to 21 days	13	28.3
22 days or more	6	13

Follow-up blood cultures

FUBCs were collected among almost all episodes of GNBSI (N=45/46, 97.8%). Only three cases (6.7%) had positive FUBC, two of the three episodes occurred in patients with ANC < 500. Among these three cases, one had *Stenotrophomonas maltophilia* of 2 days duration, which was attributed to his chemo port. Cleared was documented on day three. This case was complicated by a five-day delay from finding a positive culture to appropriate antibiotic administration. Notably, blood cultures cleared even while the patient was not on the appropriate antibiotic therapy. This patient was initially treated with intravenous trimethoprim/sulfamethoxazole and transitioned to oral minocycline for the 14-day course, with appropriate clinical response. The second patient had polymicrobial bacteremia with *Escherichia coli* and *K. pneumoniae* for two consecutive days without an unclear source. This patient was treated with cefepime with clearance of blood cultures documented on day four; however, six days following initial positive blood cultures, the patient developed Pseudomonas aeruginosa bloodstream infection from an unclear source which was cefepime-resistant and died. The final patient had polymicrobial bacteremia with *E. coli* and carbapenem-resistant *K. pneumoniae* due to a biliary source. The duration of bacteremia was two days; clearance was documented with a third set of blood cultures on day four, which were obtained following the placement of a biliary drain for source control. This patient was treated with meropenem/vaborbactam for the 14-day course, although had a five-day delay in initiating appropriate antibiotics while awaiting sensitivities.

Treatment and outcomes

Most patients were placed on appropriate initial antibiotics (N=40/46, 87.8%) when blood cultures showed Gram-negative organisms. Twenty-four cases were treated with beta-lactams (52.2%) and the most common duration was 8-14 days (N=24/47, 52.2%). Ten patients were admitted to the ICU (21.7%). ICU indications included vasopressor support (N=8/46, 17.4), mechanical ventilation (N=2/46, 21.7%), and two patients who were upgraded to the ICU for sepsis who did not require vasopressor support. Median hospital length of stay was 12 (IQR 15) days. Four patients died during their hospital stay (8.7%), while nine (19.6%) and eleven (23.9%) patients died 30 and 90 days after discharge, respectively. Readmissions were common, occurring in 21 cases (45.7%) 30 days and 24 cases (52.2%) 90 days after discharge (Table 3).

**Table 3:** Outcomes of GNBSI events (N=46) GNBSI: Gram-negative bacterial bloodstream infections

	N	%
Complications		
Admission to ICU	10	21.7
Mechanical ventilation	2	4.3
Vasopressor use	7	15.2
Mortality		
Died inpatient	4	8.7
Within 30 days of discharge	9	19.6
Within 90 days of discharge	11	23.9
Readmission		
Within 30 days of discharge	21	45.7
Within 90 days of discharge	24	52.2

## Discussion

We sought to assess the yield of FUBC among patients with hematological malignancies who have GNBSI. In our small cohort of 45 patients, we found few cases (N=3, 6.7%) of persistently positive blood cultures. Considering the current shortage of blood culture bottles, clinicians should limit the use of FUBC collection, even among patients with hematological malignancies with GNBSI, unless there is clinical suspicion of ongoing infection.

While the role of FUBC in GNBSI remains controversial, most patients (97.8%) in our cohort had FUBC collected, which is similar to prior studies among immunocompromised hosts, including solid organ and hematopoietic stem-cell transplantation recipients and patients with hematologic malignancies [7]. The utility of FUBC has been supported by prior research demonstrating a mortality benefit in collecting FUBC among patients with GNBSI [4,5,8,9]. While these prior studies have not specifically studied the benefit in patients with hematologic malignancies, many have included immunocompromised patients, including patients with malignancy and a history of transplant [4,5,8,9]. Giannella et al. found that collection of FUBCs was associated with 30-day mortality in a single Italian tertiary medical center; including some immunocompromised patients (21.1%) consisting of patients with ANC <500/μL, and solid organ or hematopoietic stem-cell transplantation [9]. A study from North Carolina among all patients at an academic medical center (including 39% of patients with a history of neoplasms) found an in-hospital mortality benefit to acquiring FUBC [4]. Amipara et al. from South Carolina found that obtaining FUBC was independently associated with reduced 28-day mortality. 16% of patients had cancer diagnosis but did not specify which patients had hematological malignancies [8]. In contrast to these prior works, Mitaka et al. did not find an association between FUBC and hospital mortality among four acute care hospitals in New York City [10]. The generalization of their findings to immunocompromised patients is limited as the authors did not specify the percentage of patients with malignancy and only included two patients with an ANC <1,000/μL [10]. The utility of FUBC among cancer patients was investigated by Clemmons and colleagues, who did not find an association with in-hospital mortality [6]. Notably, half of the cohort (N=25/52, 48%) had hematological malignancies [6]. More recently, two studies from a tertiary care hospital in Minnesota identified few positive FUBC (9-12%) in patients with hematologic malignancies, including hematopoietic stem-cell transplant recipients [11,12]. Given the unclear role of FUBC in GNBSI, identifying high-risk groups who would most benefit from repeat blood cultures should be further investigated. Ranganath et al. found that both the presence of vascular catheters and multi-drug resistant (MDR) GNBSI were associated with a high risk of repeat FUBCs, suggesting this subset of patients may benefit most from FUBCs [11]. These findings are in line with our study, as persistent bacteremia occurred in patients with MDR pathogens and indwelling central lines. Additionally, Maskarinec and colleagues developed a risk-scoring system, the AIMS score, based on clinical characteristics to help identify patients at risk for persistent GNBSI [4]. However, its applicability to patients with hematologic malignancies is unclear and needs further investigation. Our cohort only included three cases (6.7%) of positive FUBC, and we were unable to assess for mortality association given our limited sample size. Given the current blood culture media shortage [13], clinicians should limit the use of routine FUBC among patients with hematological malignancies and GNBSI [14]. Further research is warranted to elucidate the role of FUBC in this patient population.

We report several limitations. First, our sample size was small, and we only found one case without FUBC. Therefore, larger sample sizes may identify which patients are more likely to benefit from acquiring FUBC in GNBSI. Second, as our study was a retrospective, it is limited by review of and documentation in the electronic medical records. Next, since our study was conducted at a single medical center, the findings may not be generalizable to other settings but may reflect similar findings at other academic urban medical centers. Furthermore, while we did not exclude non-neutropenic patients, 84% of patients had ANC <500 cells/µL at the time of blood culture collection. Additionally, as we did not include hematopoietic stem-cell transplant recipients, these results may not be applied to transplant recipients.

## Conclusions

Positive FUBCs were infrequent (6.7%) in our cohort of GNBSI among patients with hematological malignancies. The benefit regarding the role of FUBC in GNBSI is unclear, but our findings suggest that repeat blood cultures may not be necessary for all patients with GNBSI and that the decision to repeat blood cultures should be individualized. Further investigation is needed to determine the utility of FUBC in immunocompromised patients, but considering the current blood culture shortage, clinicians should be mindful when considering repeating FUBC among patients with hematological malignancies and GNBSI. Future guidelines for GNBSI may include avoiding FUBC among all patients with hematologic malignancies.
